# Machine Learning Algorithm-Aided Determination of Predictors of Mortality from Diabetic Foot Sepsis at a Regional Hospital in South Africa During the COVID-19 Pandemic

**DOI:** 10.3390/medicina60101718

**Published:** 2024-10-20

**Authors:** Carlos Matsinhe, Shingirai Brenda Kagodora, Tshifhiwa Mukheli, Tshepo Polly Mokoena, William Khabe Malebati, Maeyane Stephens Moeng, Thifhelimbilu Emmanuel Luvhengo

**Affiliations:** 1 Department of Surgery, Thelle Mogoerane Hospital, University of the Witwatersrand, Johannesburg 2017, South Africa; carlos.matsinhe@gauteng.gov.za; 2 Department of Nuclear Medicine, University of the Witwatersrand, Johannesburg 2017, South Africa; brenda.kagodora@wits.ac.za; 3 Directorate of Oral Health and Therapeutic Services, Gauteng Province Department of Health, Johannesburg 2001, South Africa; tshifhiwa.mukheli@gauteng.gov.za; 4 Department of Podiatry, Charlotte Maxeke Johannesburg Academic Hospital, Johannesburg 2193, South Africa; polly.mokoena@gauteng.gov.za; 5 Nursing Department, Charlotte Maxeke Johannesburg Academic Hospital, Johannesburg 2193, South Africa; william.malebati@gauteng.gov.za; 6 Department of Surgery, Charlotte Maxeke Johannesburg Academic Hospital, Johannesburg 2193, South Africa; maeyane.moeng@wits.ac.za

**Keywords:** diabetic foot sepsis, COVID-19, HIV, machine learning, mortality

## Abstract

*Background and Objectives*: Diabetic foot sepsis (DFS) accounts for approximately 60% of hospital admissions in patients with diabetes mellitus (DM). Individuals with DM are at risk of severe COVID-19. This study investigated factors associated with major amputation and mortality in patients admitted with DFS during the COVID-19 pandemic. *Materials and Methods*: Demographic information, COVID-19 and HIV status, clinical findings, laboratory results, treatment and outcome from records of patients with diabetic foot sepsis, were collected and analysed. Supervised machine learning algorithms were used to compare their ability to predict mortality due to diabetic foot sepsis. *Results*: Overall, 114 records were found and 57.9% (66/114) were of male patients. The mean age of the patients was 55.7 (14) years and 47.4% (54/114) and 36% (41/114) tested positive for COVID-19 and HIV, respectively. The median c-reactive protein was 168 mg/dl, urea 7.8 mmol/L and creatinine 92 µmol/L. The mean potassium level was 4.8 ± 0.9 mmol, and glycosylated haemoglobin 11.2 ± 3%. The main outcomes included major amputation in 69.3% (79/114) and mortality of 37.7% (43/114) died. AI. The levels of potassium, urea, creatinine and HbA1c were significantly higher in the deceased. *Conclusions*: The COVID-19 pandemic led to an increase in the rate of major amputation and mortality in patients with DFS. The in-hospital mortality was higher in patients above 60 years of age who tested positive for COVID-19. The Random Forest algorithm of ML can be highly effective in predicting major amputation and death in patients with DFS.

## 1. Introduction

Diabetic foot sepsis (DFS) is among the leading causes of hospital admission in individuals with diabetes mellitus (DM) [1,2]. The two most feared complications of DFS are major amputation and death [1,2,3,4,5,6]. An amputation of the lower extremity is considered major if it is proximal to the ankle joint [2,3,4]. Around 20% of patients admitted with DFS end with a major amputation, and the in-hospital mortality is around 3–7% [2,4]. However, the mortality due to DFS is higher in patients with co-morbidities like hypertension, coronary artery disease and chronic kidney disease [1,7,8]. A state of chronic low-grade systemic inflammation from the ongoing release of pro-inflammatory cytokines is characteristic of DM [9]. Individuals with DM are likely to be overweight or obese, hypertensive and have coronary artery disease, all of which increase the risk of post-operative complications like surgical site infection (SSI) and acute kidney injury (AKI) [10].

Individuals with DM are at high risk of severe COVID-19, and among the features of severe COVID-19 disease is the over-exuberant systemic inflammatory response, the so-called “cytokine-storm”, which adds to a state of chronic low-grade inflammation that is prevalent in individuals with DM [4,11,12]. The risk of severe COVID-19, and its associated mortality, is higher in individuals older than 60 years of age who are obese or overweight with or without co-morbid conditions like CKD and DM [9,13,14,15,16].

The rise of COVID-19 cases led to the implementation of various lockdown stages, which limited movement in some countries, including South Africa [17]. Access to healthcare establishments for emergency surgical services was limited [17]. Routine pre-operative COVID-19 PCR testing became mandatory for patients who required surgical intervention at some of the hospitals. Some hospitals assigned one theatre for surgery on all patients who tested positive for COVID-19 and required emergency surgery regardless of the acuity or severity of the illness. Patients who required debridement or amputation of DFS were often not prioritised because DFS was regarded as not immediately life-threatening. However, treatment of DFS is time-sensitive, and delay in the initiation of treatment often leads to the spread of the infection and an increased possibility of major amputation or death.

The severity and, thus, likelihood of major amputation and mortality in patients with DFS, is sometimes difficult to predict even when classification systems are used [18,19,20]. Recent studies have shown the beneficial role of artificial intelligence (AI) in diagnosis, classification, treatment planning, and prediction of outcomes in patients with DFU and DFS [21,22,23]. Machine learning (ML) is the most basic form of AI. Machine learning algorithms may be supervised or unsupervised. The commonly used ML algorithms include Random Forest, Support Vector Machine and K-Nearest Neighbour for classification of categorical variables, whereas Linear Regression, Decision Tree Regression and Support Vector Regression are for continuous variables [24]. This study investigated the rate of major amputation and mortality in patients with DFS during the COVID-19 pandemic. Subsequently, the ability of ML algorithms to predict major amputation and death was studied.

## 2. Materials and Methods

This was a retrospective review of records of patients who were admitted and treated for DFS at a regional hospital in the Ekurhuleni District of Gauteng Province of South Africa from 1st March 2020 to 30th October 2021. Participants were identified using hospital admission records, and data were retrieved from in-hospital, theatre and laboratory findings. Extracted data were entered in an Excel spreadsheet. Reviewed records were limited to the period from admission until death or discharge. Records of consecutive patients who were 18 years or older and were admitted and treated for DFS were included, but patients who required re-admission were excluded.

Data retrieved included demography, site and severity of DFS, co-morbidities, HIV and COVID-19 status, laboratory results, type of treatment, level of amputation and outcome. Blood test results retrieved included haemoglobin level, white cell count (WCC), platelet count, C-reactive protein (CRP), potassium, urea, creatinine and glycosylated haemoglobin (HbA1c) levels. Post-operative complications and types of definitive treatment, including the level of amputations, were also captured. Demography and clinical findings were obtained from patients’ admission records and theatre notes and blood test results from laboratory records. The main outcome measures were major amputation and mortality. The main exposure variable was COVID-19 status and potential confounders were age, gender, HIV status and the level of haemoglobin and HbA1c. The effect modifiers included occurrence of post-operative complications including pneumonia, surgical site infection and acute kidney injury.

The reported in-hospital mortality of patients admitted and treated for DFS ranges from 3 to 7%. For this study, the predicted mortality rate for patients with DFS and who were COVID-19 PCR negative was 10% compared to an estimate of 20% for positive participants. The estimated overall sample size for a two-sample proportions with alpha set at <0.05, power at 80% and odd’s ratio of mortality of 2.25 in positive participants was 437. It was estimated that 10% of the records would be incomplete. The overall sample size was, therefore, 486. With 1:1 allocation, the net sample size for each arm was 243 participants. Approximately 120–150 with DFS are admitted annually to the hospital. The study was planned to review records of patients with DFS seen over a 4-year period from April 2020–March 2024. However, the number of COVID-19 cases reduced significantly from the middle of 2022.

### Data Analysis

The STATA© Statistics and Data Science 17.0 Standard Edition statistical package was used for the statistical analysis. Actual numbers and percentages were used to summarise categorical data, which included gender, HIV and COVID-19 statuses, types of amputation, post-operative complications and the overall outcome. The association between the overall outcome and each of the categorical variables was tested using the two-sample proportions Pearson’s chi-squared test or Fisher’s exact test, with the latter used when the count for one of the variables was less than 5%. We summarised the continuous data using either the mean with standard deviation or median and interquartile range (IQR) if the data were parametric or non-parametric, respectively. Normally, the distribution of continuous data was tested using the Shapiro–Wilk test.

Comparisons of two group means or medians for age and blood levels of potassium, haemoglobin and HbA1c of two groups of participants used the two-sided independent t-test as they were normally distributed. We used the Kruskal–Wallis test to compare medians of WCC, CRP, platelet count, urea and creatinine of the two groups of patients. The Statistical significance was set at a *p*-value below 0.05. A multivariate logistic regression was carried out to evaluate factors that had a compounding effect on mortality due to DFS in patients who had COVID-19 and adopted the purposeful selection method to determine the variables to include in multivariate logistic regression [18]. The cut-off used for inclusion in the logistic regression was a *p*-value below 0.26 following univariate analysis and less than 0.1 following multivariate logistic regression. We determined the odds of mortality with each unit change in the variables and reported it together with a 95% confidence interval. Subsequently, eight supervised ML algorithms were used to determine factors that had a correlation with mortality. 

The eight supervised ML used included Logistic Regression (LG), Naïve Bayes (NB), Decision Trees (DT), Random Forest (RF), Artificial Neural Network (ANN), Support Vector Machine (SVM), AdaBoost and CN2 Rule Induction. Additional analysis was conducted following the setting of maximum depth of each decision tree of the RF a 4 through to 11, making it RF4, RF5, RF6, RF7, RF8, RF9, RF10 and RF11. Sigmoid curves were constructed to compare the performance of the RF models. Factors that were significantly associated with mortality were matched and correlated using the ML algorithms. The performance of the ML algorithms was compared using the area under the curve (AUC) of receiver operating characteristic curves. Finally, the Kaplan–Meier survival curve to com-pare the time to in-hospital mortality between COVID-19 negative and positive participants. 

## 3. Results

There were 114 records for review; 48 (42.1%) were of female patients. The mean (SD) age of the entire group was 55 (14) years. Leg sepsis was the most prevalent diagnosis; 79 (69.3%) and right laterization 70 (61%). There were 54 (47.4%) who tested positive for COVID-19 and 41 (36%) were HIV positive. The majority—42 (36.8%) had BKA surgery, a major amputation was conducted in 79 (69.3%), and 43 (37.7%) of the patients admitted with DFS died. The median hospital stay was 11 (6–16) days. Pneumonia and SSI developed in 63 (55.3%) and 64 (56.1%), respectively. Of the 41 patients who tested positive for HIV, 56.1% (23/41) were males. Fifty-four (47.4%:54/114) patients tested positive for COVID-19, 53.7% (29/54) of whom were males. The mean (SD) age of patients who tested positive for COVID-19 was 56 (12.3) years compared to 55.5 (15.7) years of the COVID-19 negative group. Concomitant HIV and COVID-19 positive status was recorded in 21.9% (25/114) of the cases. Blood test results showed median (IQR) haemoglobin of 10.3 (9.1–12.1) g/dl, HbA1c 11.2 (9.2–12.6) and potassium 4.6 (1.2–5.4), WCC was 15 (11.9–20.1), CRP 168 (86–238), platelets 401 (260–559), urea 7.8 (4.6–14.2) and creatinine 92 µmml/L (63–192) (Table 1).

One-hundred and twelve (98.2%: 112/114) patients had surgical intervention, comprising amputations in 86.8% (97/112) and debridement in 11.4% (13/112). Thirty-seven (32.5%: 37/114) of patients ended up with an above knee intervention while 36.8% (42/114) had below knee amputation, for a major amputation rate of 69.2% (79/114). Sixty-three (55.3%: 63/114) and 26.3% (30/114) of the patients developed pneumonia and acute kidney, respectively, during admission (Table 2).

The median (IQR) length of stay of all the patients was 11 (7–17) days. Forty-three (37.7%:43/114) of the patients died, 62.8% (27/43) of whom were males. However, the influence of gender on mortality was not statistically significant (*p*-value = 0.410). The patients who died were significantly older, at a mean (SD) age of 60.3 (15.5) years, compared to the mean age of 53 (12.6) years for those who were discharged (*p*-value = 0.007). Other parameters that were statistically, and, thus, significantly different in patients who died, compared to those who were discharged, included HIV positive status of 37.2%, versus 19.7% COVID-19 infection rate of 83.7%, versus 25.4% and higher serum levels of potassium (Table 3).

Fifteen (34.9%) of the patients who died had AKA compared to 31% of those who got discharged. The difference in treatment options between patients who died and those who were discharged was not statistically significant (*p*-value = 0.178). Similarly, the difference in the rate of major amputation of 79.1% and 63.4% in patients who died and those who died was not statistically significant (*p* = 0.78). However, differences in the rate of occurrence of SSI (*p*-value < 0.001), AKI (*p*-value < 0.001), pneumonia (*p*-value < 0.001) and UTI (*p*-value < 0.001) between patients who died and were discharged were statistically significant (Table 4).

Although the lower level of platelet count (*p*-value = 0.400) and the raised serum potassium (*p*-value = 0.108), urea (*p*-value = 0.591) and creatinine (*p*-value = 0.653) were significantly associated with mortality in the univariate analysis, their influence diminished following a multivariate analysis, unlike older aged and concurrent COVID-19 and HIV infection(s) (Table 5).

Among those who died, the mean/median was: 60 (15) years for age, 9.8 (8–11.3) for haemoglobin level, 315 (201–559) for platelets, 5.1 (4.4–5.9) for potassium, 13 (5.6–21.2) for urea, 176 (66–302) for creatinine; and the mean/median length of their hospital stay was 7 (5–16) days. Of those who died, 36 (84%) had positive COVID-19 results, and 27 (63%) were HIV positive. In relation to the information gain of our model, the following variables were the top five predictors for the outcome of death or discharge: pneumonia, COVID-19, SSI, AKI and creatinine with information gain of 0.333, 0.248, 0.195, 0.166 and 0.15, respectively.

Based on the data obtained, several models were evaluated to predict an outcome of death or discharged in patients with DFS. The models were assessed using various metrics, including Area Under the Receiver Operating Characteristic (AUC under the ROC curve), Classification Accuracy (CA), F1 Score, Precision, Recall and Matthews Correlation Coefficient (MCC). The RF model demonstrated exceptional performance with an AUC of 0.965, a CA of 0.895, an F1 score of 0.893, and a high MCC of 0.775. These metrics suggest that the RF model has a strong ability to predict outcomes accurately, with balanced precision and recall.

Gradient Boosting also performed well, achieving an AUC of 0.947, a CA of 0.886, and an F1 score of 0.885. However, it was slightly less effective than the RF model, as indicated by its lower MCC of 0.755. Similarly, the Naïve Bayes had a commendable performance with an AUC of 0.931 and an F1 score of 0.868. However, it still lagged the RF model. Both the RF and Naïve Bayes fared much better compared to Logistic Regression, SVM and Neural Network. Logistic Regression, SVM, and Neural Network had significantly lower AUC values, like 0.478 of Logistic Regression, indicating a poor predictive capability for the given dataset (Table 6).

The ensemble technique, referred to as “Stack”, outperformed all individual models, achieving the highest AUC of 0.966, CA of 0.904, and an F1 score of 0.903. Its MCC of 0.793 also indicates strong performance and generalizability. The RF model was tested across multiple folds from RF4 to RF11 and showed consistent results throughout. The AUC remained around 0.970, with a CA of 0.895 and an F1 score of 0.893. The consistency of these results across different folds confirms the robustness of the RF model (Table 7).

**Figure 1:**
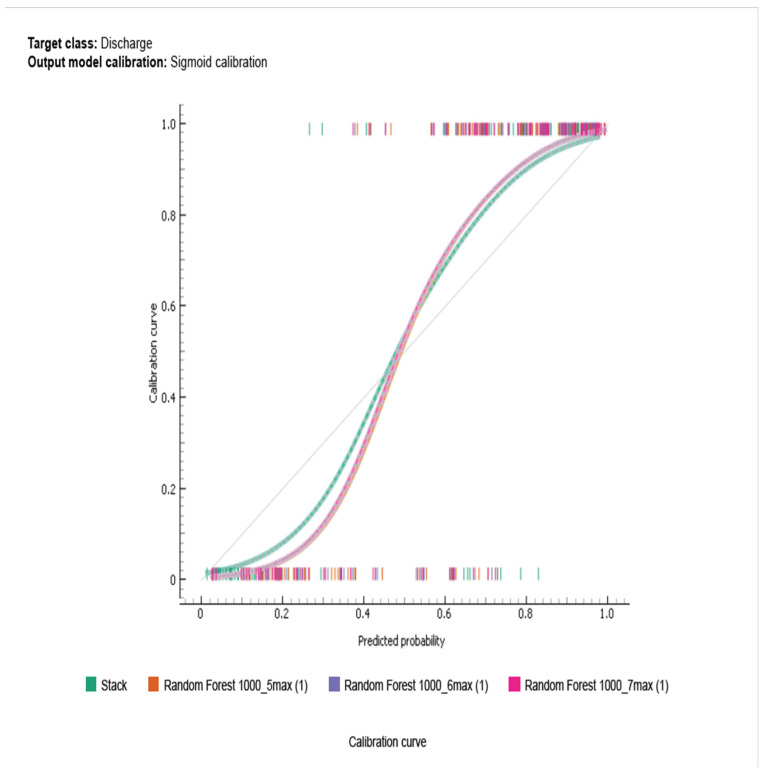
Comparison of the sigmoid curves of RF5 max, RF6 max and RF5 max depicting the more favourable performance of RF5 Max.

Among all the models tested, the RF Stack ensemble technique performed the best, with the highest overall metrics across the board. However, when focusing on individual models, RF5 provided the most reliable performance, particularly in terms of AUC and MCC. These findings indicate that ensemble methods (Stack) or RF can be highly effective in predicting clinical outcomes in DFU patients, offering reliable and robust predictions. The final models with a favourable F1 score of 0.919 was RF5 with an AUC of 0.967, CA of 0.895, PREC of 0.883, recall of 0.958 and MCC of 0.775. Figure 1 compares the sigmoid curves of RF5 max, RF6 max and RF5 max. 

**Figure 2:**
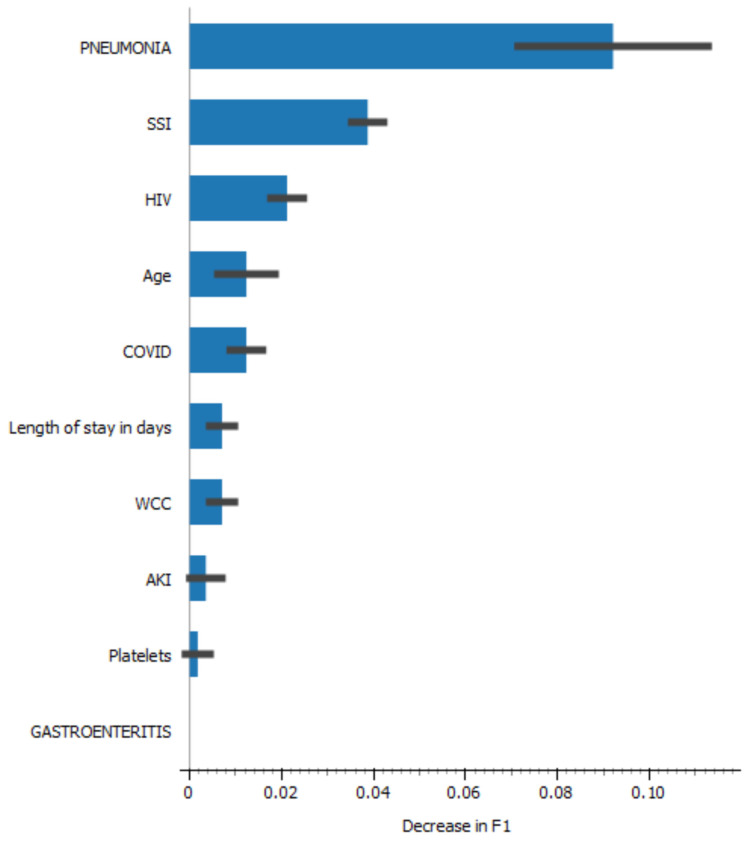
Ranking of predictors of death in patients with DFS (N = 114). SSI = surgical site infection; HIV = human immunodeficiency virus; WCC = acute kidney injury.

Pneumonia and surgical site infection were stronger predictors of mortality (Figure 2). 

**Figure 3:**
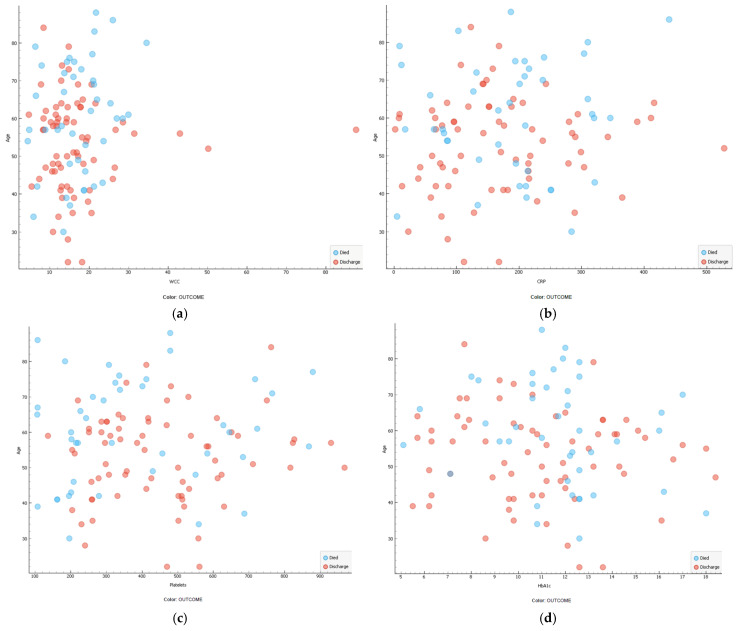
(**a**) Scatter plots comparing the correlation of age in years and WCC between patients who died and those who were discharged (N = 114). (**b**) Scatter plots comparing the correlation of age in years and CRP between patients who died and those who were discharged (N = 114). (**c**) Scatter plots comparing the correlation of age in years and platelet count between patients who died and those who were discharged (N = 114). (**d**) Scatter plots comparing the correlation of age in years and platelet count between patients who died and those who were discharged (N = 114).

In comparison to the age of different biomarkers for different outcomes, the scatterplots show variations. A WCC < 30 × 10^9^/L was in older patients who died, and CRP in older patients had a wide scatter range, while platelet counts were lowest in the majority of patients who died. The scatter plots indicating the correlation of age and HbA1c range show that more scatter is observed in ages over 50 years. For scatter plots showing the comparison of correlation age versus WCC, CRP, platelet count, and HbA1c, is shown in Figure 3a–d. 

**Figure 4:**
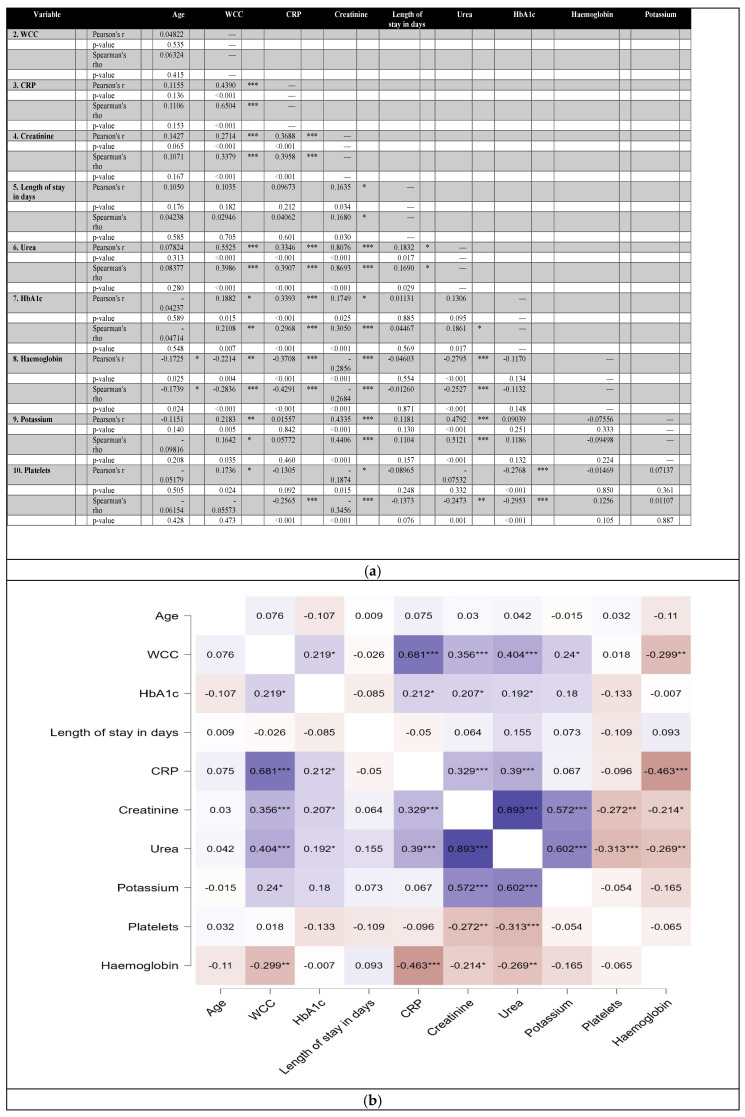
(**a**) Correlation among haematological, biochemical results and length of hospital stay. *, ** and *** are when *p*-value is <0.05, <0.01 and <0.001, respectively. (**b**) Heat-map depicting results of correlation analysis among haematological, biochemical results and length of hospital stay. Darker colours depict strong positive (blue) or negative (brown) correlation. Strong positive correlation is closer to +1 whereas strong negative is closer to −1. *, ** and *** depict strong, moderate and very strong negative or positive correlation.

Most patients died or were discharged within 20 days; the majority of those who died did not exceed 15 days across all age ranges. There was a strong positive correlation for urea and creatinine r = 0.893, CRP and WCC r = 0.681, and urea and potassium r = 0.602 (Figure 4a,b).

**Figure 5:**
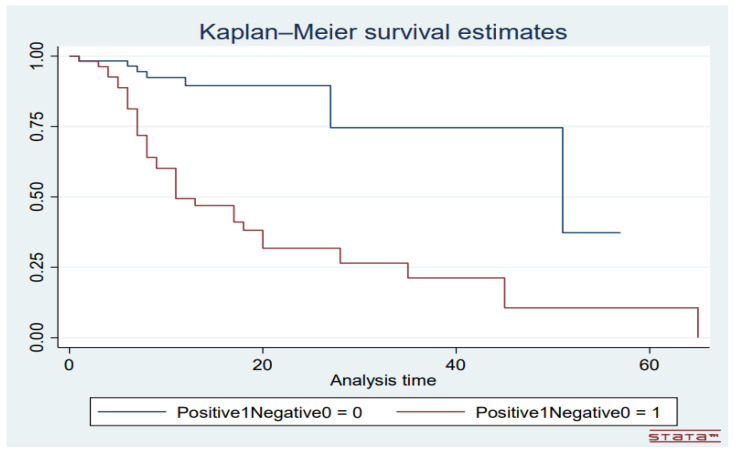
Kaplan–Meier curve comparing time to death in days between COVID-19 negative (blue line) COVID-19 positive (red line) with DFS observed over 1652 person-days. *Y*-axis depicts mortality rate and *X*-axis the length of stay in hospital before either death or discharge.

The median length of hospital stay of patients was statistically significantly shorter than that of those who were discharged (*p* = 0.018). The length of hospital stay of the patients was however not influenced by gender (*p* = 0.2800) or COVID-19 status (*p* = 0.492). Majority of the deaths in patients who tested positive for COVID-19 occurred within the first 10 days following admission to the hospital (Figure 5).

## 4. Discussion

Diabetic foot sepsis is responsible for the majority of hospital admissions of individuals with DM. Measures of outcome following admission of a patient include, the number of debridement, the need and level of amputation, length of hospital stay and the 30-day mortality [5,6]. This study set out to investigate the influence of the COVID-19 pandemic on the rate of major amputation and in-hospital mortality in patients who had DFS. Among the main findings, there was a high rate of COVID-19 infections in patients admitted with DFS, a major amputation rate of 70% and an overall mortality rate of 38%. Additionally, most of the patients who died were above the age of 60 years, and 84% of those who died were patients who tested positive for COVID-19.

Complications of DM, including DFS, were more common in men as the current study demonstrated [25]. Furthermore, more men with DFS tested positive for COVID-19, which is in line with previous findings [3,26]. Among the plausible explanations for the high rate of COVID-19 infection and mortality in men is a higher density of angiotensin-converting enzyme receptors 2 (ACE2) in their hearts, lungs, kidneys, gastrointestinal tract and blood vessels, compared to women [27,28]. Furthermore, men often delay seeking treatment, which might have also contributed to higher mortality [6].

The mean age of the patients in the current study was 59 years, which is like findings from studies conducted in other countries [3,4,6]. The mortality rate in our study was highest in patients who were older than 60 years, which was not surprising as the elderly are likely to have co-morbidities like coronary artery disease, hypertension and chronic kidney disease that increase the likelihood of post-operative death [14,26]. Some of the patients who concurrently were hypertensive might have been on ACE inhibitors. The use of ACE inhibitors can cause the up-regulation of ACE2 receptors, which increases the risk of COVID-19 infection, and the development of severe disease [11]. The likelihood of severe COVID-19 and its complications, including death, is higher in individuals above the age of 60 [11,25].

Patients with DFS may be known or previously unknown with DM [8]. Regardless of their background history, HbA1c in individuals with DM complicated by DFS is usually above 7.5%, as was the case for most patients in the current study [7]. Serum levels of potassium, urea and creatinine in the patients who died were markedly higher than in those who survived, which was not surprising as a combination of severe sepsis due to DFS and the cytokine storm of COVID-19 increases the risk of acute kidney injury [9,14,15]. The likelihood of acute injury is especially higher in individuals who are already at an increased risk of renal dysfunction, like patients with DM [9,14,15]. The low platelet count levels observed in patients who died are consistent with the expectations in severe sepsis and the cytokine storm of COVID-19.

The key priorities during the management of a patient with DFS include fluid resuscitation, correction of electrolyte derangements, glycaemic control and early initiation of treatment with a broad-spectrum antibiotic [20]. Acute kidney injury is among the common complications of DFS, as demonstrated in the study [14]. Acute care of a patient who has DFS is, however, labour-intensive and requires hourly monitoring, which might not have been feasible during the COVID-19 pandemic [27]. Debridement and/or amputation of DFS is conducted in theatre following fluid resuscitation, and when a patient’s hydration status, electrolyte derangements and acid-base status have been corrected, as well as when their level of blood glucose is below 15 mmol/L [4,8]. The need for amputation depends on the severity of the infection, and patients with spreading necrotizing infection or wet gangrene require a guillotine amputation [4].

Of concern to the current study was the 70% rate of major amputation, which was much higher than the 12.9% reported by Aulivola et al. [26]. Although high, the rate of major amputation in our study mirrors the findings by Cheddie and colleagues in a study carried out in KwaZulu-Natal in South Africa [3]. What was more concerning was the 47% of major amputations that were above the knee. Interestingly, neither concurrent HIV nor COVID-19 infection had an influence on the rate of major amputation, which is contrary to the findings by Chaudhary et al., 2021 [4] and Zayed et al., 2022 [16].

Patients with DM are immunocompromised and prone to local and systemic post-operative complications. The current study only focused on the final amputation and did not document the overall number of procedures conducted in each patient. The most common local complication following amputation for DFS is SSI, which often necessitates “salami” amputations [7]. The other complications that are common in patients admitted for management of DFS with or without COVID-19 are pneumonia and acute kidney injury, which was also the case in the present study [3,8]. The overall mortality in the study was 38%, which is four times higher than the 8.6% reported before the COVID-19 pandemic [20]. The majority—84% of the deaths in patients with DFS was among patients who tested positive for COVID-19, which conforms with the high rate of severe COVID-19 and mortality in individuals with DM [11]. Most deaths in the current study were in individuals above the age of 60, which is consistent with previous findings [6]. Additionally, most of the mortalities occurred in the participants who had a major amputation, which aligns with findings from previous studies [7,28,29,30].

Recent studies have shown the beneficial role of artificial intelligence (AI) in the diagnosis, classification, treatment planning, and prediction of outcome in patients with DFU and DFS [21,22,23,24]. Machine learning (ML) is the most basic form of AI. Machine learning algorithms may be supervised or unsupervised. The commonly used ML algorithms include Random Forest, Support Vector Machine and K-Nearest Neighbour for the classification of categorical variables, whereas Linear Regression, Decision Tree Regression and Support Vector Regression are for continuous variables [22,24]. The RF model demonstrated exceptional performance with an AUC of 0.965, and a strong ability to predict outcomes accurately, with balanced precision and recall.

Although there are a limited number of studies on the role of ML-aided screening, diagnosis and decision-making in the management of DFS, there is a lot written on the role of AI in DM [31,32]. The use of AI for the screening of DM-associated peripheral neuropathy has grown exponentially [31]. Peripheral neuropathy is the most common complication in patients with DM and is the major risk factor for DFU [31]. Most patients with DFS had DFU, which did not heal. Once a neuropathic or neuro-ischaemic DFU has developed, the focus should be on expediting healing and preventing the development of DFS. Patients with DFS are likely to end up with a major amputation and subsequently die within 5 years [33].

Patients with DFS require timeous and aggressive treatment to prevent amputation or death. Knowing which patients most at risk of death are is important for tailoring of the aggressiveness of the treatment. In a study by Stefanopoulos et al., using ML-algorithm ages above 40, gangrene, septic shock, low haemoglobin levels, and anaemia were among the factors that contributed significantly to the need for major amputation during admission [34]. Similarly, following the application of ML predictive models, Xie et al. (2021) found that patients with DFS had elevated WCC and serum creatinine during admission; among other factors, this increased the likelihood of them ending up with a major amputation [35]. Our study found that pneumonia, COVID-19, SSI, AKI and creatinine are strongly associated with mortality in patients with DFS. A study by Radunovic et al. (2023) combined categorical and continuous variables and found that among others, the age of a patient, haemoglobin levels, urea, creatinine, glomerular filtration rate and length of hospital stay were significantly associated with mortality from DFS [31]. However, the aim of the above study was to determine the survival rate of patients with DFU over a 5-year and 10-year period.

This study was retrospective; it is likely that we missed some records. The sample size is relatively small, and, thus, the sample was not divided into training, testing and validation set. A small sample size might have led to over-fitting. We did not investigate the influence of potential compounding factors, such as the expertise involved in treatment decisions and waiting time for theatre, and the study was based at one facility. Therefore, the findings may not be generalizable.

## 5. Conclusions

The COVID-19 pandemic led to an increase in the rate of major amputation and mortality in patients with DFS. COVID-19 status had no influence on the rate of amputation. In-hospital mortality was higher in patients who were older than 60 and tested positive for COVID-19. We recommend prioritising patients with DFS for aggressive and timeous treatment during a pandemic, as they are at a high-risk of major amputation and mortality.

## Figures and Tables

**Table 1:** Demography, extent of sepsis and laboratory of patients with DFS (N = 114).

Variable	Results
Gender	
Male	66 (57.9%)
Female	48 (42.1%)
Age (SD)	55(14) years
Diagnosis	
Cellulitis	1 (0.9%)
Foot sepsis	26 (22.8%)
Gangrenous foot	1 (0.9%)
Leg sepsis	4 (3.5%)
Leg ulcer	3 (2.6%)
Leg sepsis	79 (69%)
Laterization	
Left	43 (37.7%)
Right	70 (61.4%)
Bilateral	1 (0.9%)
Laboratory results	
WCC (IQR)	15 (11.9–20.1) × 10^9^/L
CRP (IQR)	168 (86–238) mg/dL
Haemoglobin (IQR)	10.3 (9.1–12.1) g/dL
Platelets (IQR)	401 (260–559) × 10^9^/L
Potassium (IQR)	4.6 (1.2–5.4) mmol/L
Urea (IQR)	7.8 (4.6–14.2) mmol/L
Creatinine (IQR)	92 (63–192) µmml/L
HbA1c	11.2 (9.2–12.6%)
COVID-19	
No	60 (52.6%)
Yes	54 (47.4%)
HIV status	
No	73 (64%)
Yes	41 (36%)

**Table 2:** Treatment options, nature of anaesthesia and in-hospital complications (N = 114).

Variable	Results
Type of Surgery	
Above knee amputation	37 (32.5%)
Below knee amputation	42 (36.8%)
Debridement	13 (11.4%)
None	2 (1.8%)
Trans-metatarsal amputation	7 (6.1%)
Toectomy	13 (11.4%)
Major amputation	
No	35 (30.7%)
Yes	79 (69.3%)
Type of anaesthesia	
General	25 (21.9%)
Spinal	87 (76.3%)
Not applicable	2 (1.8%)
Outcome	
Died	43 (37.7%)
Discharged	71 (62.3%)
Length of hospital stay (IQR)	11 (6–16) days
Surgical site infection	
No	53 (46.5%)
Yes	61 (53.5%)
Acute kidney injury	
No	84 (73.7%)
Yes	30 (26.3%)
Pneumonia	
No	51 (44.7%)
Yes	63 (55.3%)
Deep vein thrombosis	
No	113 (99.1%)
Yes	1 (0.9%)
Urinary tract infection	
No	82 (71.9%)
Yes	32 (28.1%)
Gastroenteritis	
No	100 (87.7%)
Yes	14 (12.3%)

**Table 3:** Comparison of demographic characteristics and laboratory results of participants with DFS based on overall outcome of death or discharge (N = 114).

Variable	Died	Discharged	*p*-Value
Gender			
Male	27 (62.8%)	39 (54.9%)	0.410
Female	16 (37.2%)	32 (45.1%)	
Age (SD)	60 (15)	53 (12.6)	0.007
Nature of involvement			
Cellulitis	0 (0%)	1 (1.4%)	0.830
Foot sepsis	11 (25.6%)	15 (21.1%)	
Gangrenous foot	1 (2.3%)	0 (0%)	
Leg sepsis	1 (2.3%)	3 (4.2%)	
Leg ulcer	1 (2.3%)	2 (2.8%)	
Leg sepsis	29 (67.4%)	50 (70.4%)	
Laterization			
Left	20 (46.5%)	23 (32.4%)	0.072
Right	22 (51.2%)	48 (67.6%)	
Bilateral	1 (2.3%)	0 (0%)	
Laboratory results			
WCC	17.2 (13.45–21.33)	14.54 (11.6–18.37)	0.0980
CRP	201 (103–251)	152 (85–221)	0.1874
Haemoglobin	9.8 (8–11.3)	10.9 (9.6–12.6)	0.0021
Platelets	315 (201–559)	417 (298–561)	0.0283
Potassium	5.1 (4.4–5.9)	4.5 (4.1–5.1)	0.0014
Urea	13 (5.6–21.2)	7.6 (4.1–11.1)	0.0038
Creatinine	176 (66–302)	89 (55–128)	0.0039
HbA1c	12.05 (10.6–12.6)	10.8 (8.6–13.2)	0.1353
COVID-19 status			
No	7 (16.2%)	53 (74.6%)	<0.000
Yes	36 (83.7%)	18 (25.4%)	
HIV status			
No	16 (37.2%)	57 (80.3%)	<0.000
Yes	27 (62.8%)	14 (19.7%)	

**Table 4:** Comparison of treatment instituted and outcomes between patients who died and those who were discharged (N = 114).

Variable	Died	Discharged	*p*-Value
Extent of surgery			
Above knee amputation	15 (34.9%)	22 (31%)	0.178
Below knee amputation	19 (44.2%)	23 (32.4%)	
Debridement	4 (9.3%)	9 (12.7%)	
None	1 (2.3%)	1 (1.4%)	
Trans-metatarsal amputation	3 (6.9%)	4 (5.6%)	
Toectomy	1 (2.3%)	12 (16.9%)	
Major amputation			
No	9 (20.9%)	26 (36.6%)	0.078
Yes	34 (79.1%)	45 (63.4%)	
Type of anaesthesia			
General anaesthesia	11 (25.6%)	14 (19.7%)	0.619
Spinal anaesthesia	31 (72.1%)	56 (78.9%)	
Not applicable	1 (2.3%)	1 (1.4%)	
Surgical site infection			
No	34 (79.1%)	19 (26.8%)	<0.0001
Yes	9 (20.9%)	52 (73.2%)	
Acute kidney injury			
No	20 (46.5%)	64 (90.1%)	<0.0001
Yes	23 (53.5%)	7 (9.9%)	
Pneumonia			
No	2 (4.7%)	49 (69%)	<0.0001
Yes	41 (95.3%)	22 (31%)	
Deep vein thrombosis			
No	43 (100%)	70 (98.6%)	1.000
Yes	0 (0%)	1 (1.4%)	
Urinary tract infection			
No	40 (93%)	42 (59.2%)	<0.0001
Yes	3 (7%)	29 (40.8%)	
Gastroenteritis			
No	36 (83.7%)	64 (90.1%)	0.311
Yes	7 (16.3%)	7 (9.9%)	
Length of hospital stay	7 (5–16)	12 (8–16)	0.0269

**Table 5:** Results following multivariate logistic regression analysis for covariates and compounders that were predictive of mortality in patients who had DFS.

Variable	Odds Ratio	Standard Error	Z	*p*-Value	95% CI
Age	1.065	0.025	2.65	0.008	1.016–1.115
Haemoglobin	0.655	0.086	−3.20	0.001	0.506–0.849
COVID-19 positive	39.718	29.352	4.98	<0.001	9.332–169.053
HIV positive	12.698	8.499	3.80	<0.001	3.419–47.153

**Table 6:** Comparison of performance of various ML algorithms for prediction of outcomes.

Model	AUC	CA	F1	Prec	Recall	MCC
Logistic Regression	0.49	0.623	0.478	0.388	0.623	0
Random Forest	0.965	0.895	0.893	0.897	0.895	0.775
Gradient Boosting	0.947	0.886	0.885	0.886	0.886	0.755
Tree	0.809	0.807	0.809	0.816	0.807	0.604
AdaBoost	0.748	0.754	0.756	0.76	0.754	0.488
SVM	0.498	0.456	0.462	0.47	0.456	−0.127
Neural Network	0.482	0.509	0.511	0.513	0.509	−0.036
Naïve Bayes	0.931	0.868	0.868	0.868	0.868	0.719
CN2 Rule Induction	0.629	0.605	0.606	0.607	0.605	0.164

**Table 7:** Comparison of results from various models of RF and with other ML algorithms.

	AUC	CA	F1	Prec	Recall	MCC
RF	0.965	0.895	0.893	0.897	0.895	0.775
Gradient Boosting	0.947	0.886	0.885	0.886	0.886	0.755
Naïve Bayes	0.931	0.868	0.868	0.868	0.868	0.719
Stack	0.966	0.904	0.903	0.903	0.904	0.793
RF4	0.968	0.895	0.893	0.897	0.895	0.775
RF5	0.971	0.895	0.893	0.897	0.895	0.775
RF6	0.971	0.895	0.893	0.897	0.895	0.775
RF7	0.971	0.895	0.893	0.897	0.895	0.775
RF9	0.97	0.895	0.893	0.897	0.895	0.775
RF10	0.97	0.895	0.893	0.897	0.895	0.775
RF11	0.97	0.895	0.893	0.897	0.895	0.775

## Data Availability

Anonymized data for the study will be made available on request.
